# Dexmedetomidine is associated with better outcomes in patients undergoing cardiac surgery

**DOI:** 10.1186/cc10934

**Published:** 2012-03-20

**Authors:** PG Brandão, S Lobo, M Nassau Machado, J Duarte, F Lobo, Y Sakr

**Affiliations:** 1 Hospital de Base de São José do Rio Preto, Brazil; 2 Friedrich Schiller University Hospital, Jena, Germany

## Introduction

Cardiac anesthesia has changed over the years from high-dose opioids to fast-track surgery. The use of high doses of opioids was justified based on the hemodynamic stability [1] at a cost of prolonged mechanical ventilation support. Our study aims to analyze the use of dexmedetomidine as an anesthesia adjuvant during the induction and maintenance of anesthesia for patients undergoing coronary artery bypass graft (CABG) and valvular heart surgeries.

## Methods

This study is a retrospective analysis from a prospective database collected from January 2003 to April 2011. The patients were divided into two groups, based on the use of dexmedetomidine (DEX group) intraoperatively or conventional opioid-based technique (Control group). Isoflurane was used for anesthesia maintenance in both groups.

## Results

We included 1,302 consecutive patients undergoing cardiac surgery during the study period (63% male; median age = 57 years), 796 patients in the DEX group and 506 patients in the control group. CABG was the most commonly performed surgery (63%) followed by valve surgeries (37%). The overall 30-day hospital mortality rate was 5.8%. Length of stay was significantly lower for patients in the Dex group (3.7 ± 4.4 days) than for patients in the control group (4.5 ± 6.3 days) (*P *= 0.02). Thirty-day mortality rates were 3.4% in the Dex group and 9.7% in the control group (*P *< 0.001). In the multivariable Cox regression analysis with in-hospital death as the dependent variable, dexmedetomidine (OR = 0.39, 95% CI: 0.23 to 0.64, *P *≤0.001), a high L-EuroSCORE (OR = 1.05, 95% CI: 1.01 to 1.10, *P *= 0.004) and older age (OR = 1.03, 95% CI: 1.01 to 1.05, *P *= 0.003) were independently related to in-hospital death. Need for reoperation (2.0% vs. 2.8%, *P *= 0.001), neurologic lesion type 1 (2.0% vs. 4.7%, *P *= 0.005) and prolonged hospitalization (3.1% vs. 7.3%, *P *= 0.001) were significantly less frequent in the DEX group than in the control group.

## Conclusion

Use of dexmedetomidine as anesthesia adjuvant was associated with better outcomes in patients undergoing cardiac surgery.
